# Vehicle Detection in Drone Aerial Views Based on Lightweight YOLOv10-IAD

**DOI:** 10.3390/s26113585

**Published:** 2026-06-04

**Authors:** Lei Zhang, Zhongmin Li, Yufeng Yao

**Affiliations:** 1 School of Information Engineering, Nanchang Hangkong University, Nanchang 330063, China; 2 School of Engineering, University of the West of England, Bristol BS16 1QY, UK

**Keywords:** vehicle detection, YOLOv10 model, involution convolution, ACmix, DyHead

## Abstract

UAV-based vehicle detection faces challenges of small targets, dense distribution, and occlusions. Built upon YOLOv10n, this paper proposes YOLOv10-IAD by integrating three modules: (1) Involution convolution in the backbone to enlarge the receptive field and enhance spatial perception for small targets; (2) ACmix (Attention and Convolution Mixed) in the neck to fuse local details with global context; (3) DyHead (Dynamic Head) that recalibrates features via scale-, space-, and task-aware attention, improving localization for occluded objects. On VisDrone2019 and UAVDT datasets, YOLOv10-IAD improves mAP50 by 3.7% (to 47.2%) and 3.5% (to 52.0%), and recall by 3.1% and 2.0%, respectively, with only a modest increase in parameters (2.9 M) and computational cost. Compared to other YOLO series, it achieves a favorable trade-off between detection accuracy and computational efficiency. These advancements make it suitable for deployment on hardware onboard UAVs for real-time road vehicle detection.

## 1. Introduction

The rapid development of unmanned aerial vehicles (UAVs) technology has provided new technical means for wider applications such as intelligent traffic monitoring, urban security management, and emergency rescue [1,2]. Compared with traditional fixed monitoring equipment, UAVs offer some unique advantages including mobility, ease of deployment, and a wide field of view, enabling large-scale real-time dynamic data collection [1]. Recent reductions in hardware cost and improvements in endurance have led to widespread UAV deployment in traffic flow monitoring, road inspection, and accident rapid response, making UAVs an increasingly important component of intelligent transportation systems [2,3].

Vehicle detection applying UAV aerial images, however, faces three major challenges that are less pronounced in general object detection: small scale (i.e., vehicles often occupy only tens of pixels), dense distribution with frequent occlusions, and complex background interference (i.e., roads, buildings, shadows, etc.) [4,5]. These characteristics demand enhanced capabilities in context modeling, multi-scale feature fusion, and dynamic adaptation.

With the continuous development of deep learning, a growing number of methods have been developed and applied to the UAV-assisted vehicle detection [6,7]. Existing approaches mainly fall into two categories: two-stage and one-stage detectors. Two-stage algorithms adopt a “region proposal + fine classification” framework, achieving high recall and precise localization [8,9]. Hansen et al. systematically compared Faster R-CNN and SSD for vehicle detection using UAV images, confirming the accuracy advantage of Faster R-CNN in terms of accuracy [10]. Darehnaei et al. proposed SI-EDTL [11], a two-stage ensemble algorithm that integrates three pre-trained feature extractors and five transfer classifiers. Despite their accuracy, two-stage detectors suffer from high computational complexity and slow inference speed, which become particularly problematic for resource-constrained UAV platforms [12]. A recent IEEE study showed that even with lightweight backbones, regression modules in advanced two-stage detectors still consume substantial computational resources, making real-time airborne processing difficult [13].

In comparison, one-stage detectors directly regress object categories and bounding box coordinates, enabling end-to-end detection and offering some significant advantages in inference speed [14,15]. To address the challenges of complex backgrounds, small-scale target sizes, and limited computational resources with UAV aerial images, various improved YOLO (You Only Look Once) algorithms, which are typical one-stage detectors, have been proposed [16,17]. Yang et al. developed YOLOv5-R, which introduces coordinate attention (CA) in the backbone, a weighted BiFPN in the neck, and a dynamic head (DyHead) to boost performance [16]. For dense small-scale target detection at high altitudes, Zhu et al. proposed YOLO-Drone, using a Darknet59 backbone and an MSPP-FPN module with spatial and atrous pyramid pooling to enhance multi-scale feature extraction [17]. Cao et al. improved YOLOv8n by adding a P2 small-scale target detection layer, introducing receptive-field attention convolution (RFAConv), and using normalized Wasserstein distance (NWD) for bounding box similarity [18]. In order to trade off complexity with accuracy, Zhou et al. proposed a lightweight method with multi-layer selective features, incorporating RFAConv, a CSP-BLRAN module, an MS-FPN neck, and a generalized Wasserstein distance loss (GWDLoss) [19].

As one of the latest YOLO series, YOLOv10 was proposed by Wang et al. in 2024, introducing a consistent dual assignment strategy for NMS-free training and a holistic efficiency–accuracy-driven model design [20]. Extensive experiments have demonstrated that YOLOv10 achieves state-of-the-art performance and efficiency across various model scales on the COCO benchmark [20]. However, directly applying its original architecture together with UAV aerial scenes reveals several limitations rooted in its design platform: First, the backbone relies on stacked standard convolutions with spatially shared kernels, which limits its ability to capture long-range dependencies for distant small-scale targets [21]. Second, the neck’s PANet structure, built upon fixed-receptive-field C2f modules, underutilizes detailed shallow features during multi-scale fusion [22]. Third, the original decoupled detection head applies static weights uniformly across all spatial locations and channels, thus lacking content-aware mechanisms to handle severe scale variations and partial occlusions [23]. These limitations restrict the performance of YOLOv10 in UAV-assisted vehicle detection applications.

To address these issues, this paper proposes an improved UAV-assisted vehicle detection model named YOLOv10-IAD by combining Involution convolution, the ACmix module and the DyHead module. The main novelties are as follows:

- Involution convolution is introduced into the backbone network. Proposed by Li et al. at CVPR 2021, Involution adopts a channel-shared, space-specific design that dynamically generates kernels based on input content, inverting the design principles of standard convolution [24]. This allows us to expand the kernel size from 3 × 3 to 7 × 7 with negligible parameter increase, significantly enlarging the receptive field and enhancing spatial perception for dense small scale targets.
- The ACmix module is embedded into the neck network. Proposed by Pan et al. at CVPR 2022, ACmix unifies self-attention and convolution by revealing that the bulk of the computations of these two paradigms are carried out with the same operation and then fuse local response aggregation and global context modeling via learnable scalars [25]. This integration improves multi-scale feature interaction efficiency while preserving both local details and global semantics.
- The DyHead module replaces the original detection head. Proposed by Dai et al. at CVPR 2021, DyHead applies three serial dynamic enhancement units—scale-aware, spatial-aware, and task-aware attention—to dynamically recalibrate features [26]. This enables the detection head to adaptively adjust its response based on input content, thereby improving localization accuracy for occluded and scale-variant targets.

The main novelty of this work lies not in the individual modules but in their synergistic integration into the YOLOv10 architecture, where each module compensates for a specific weakness of the baseline while the combination yields positive interactions (quantified in Section 3.3.2). To the best of our knowledge, this is the first attempt to jointly optimize YOLOv10 for UAV detection using this specific triplet of modules, and we provide a systematic ablation and synergy analysis that reveals why ACmix outperforms BiFPN in this context.

## 2. Methodology

### 2.1. YOLOv10 Algorithm

**Figure 1:**
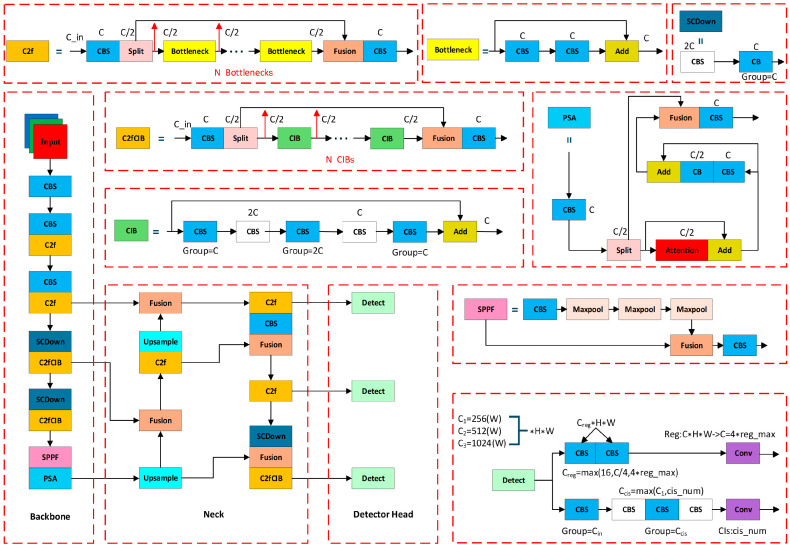
The overall network architecture of YOLOv10.

YOLOv10 serves as the baseline model in this study, and its architecture is illustrated in Figure 1. Proposed by a research team from Tsinghua University in 2024, YOLOv10 aims to resolve the post-processing dependency and architectural inefficiencies inherited from previous YOLO series. Building upon the efficient architecture of YOLOv8, YOLOv10 introduces a dual-label assignment strategy centered on the elimination of Non-Maximum Suppression (NMS), fundamentally addressing the inference latency caused by post-processing in traditional YOLO detectors. Emphasizing a balance between overall efficiency and accuracy, YOLOv10 replaces the C2f module in the backbone with a rank-guided Compact Inverted Block (CIB) and employs spatial-channel decoupled downsampling (SCDown) to separate spatial reduction from channel modulation, thereby minimizing information loss and computational cost. Furthermore, the detection head of YOLOv10 adopts a dual-head design, enabling efficient NMS-free end-to-end deployment.

### 2.2. YOLOv10-IAD Network Model

**Figure 2:**
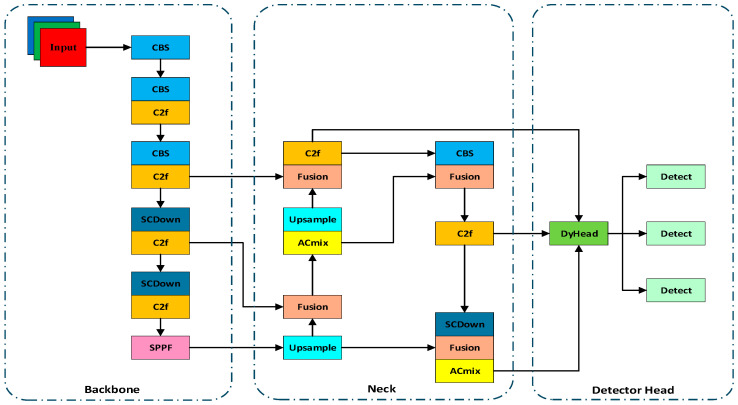
The structure of the YOLOv10-IAD model.

To achieve better vehicle detection using UAV aerial imagery, we focused on common challenges in this task, such as small target scale, dense distribution, frequent occlusion, complex backgrounds, and high miss rates. By conducting an in-depth analysis of these issues, we propose our YOLOv10-IAD model, optimized for UAV-assisted vehicle detection based on YOLOv10n, as shown in Figure 2. We introduced Involution into the backbone to expand the receptive field and enhance spatial perception for dense small targets. We embedded ACmix into the neck to fuse local details with global context, improving multi-scale feature interaction. Additionally, we replaced the detection head with DyHead, which dynamically recalibrates features via scale, spatial, and task attention, improving localization accuracy for occluded and scale-variant targets. These improvements enable YOLOv10-IAD to achieve a superior balance between detection accuracy and computational efficiency within complex UAV aerial scenes.

#### 2.2.1. Feature Extraction Network

The feature extraction of the YOLOv10 model mainly relies on the C2f modules in its backbone. The C2f module achieves lightweight design through feature reuse and branching structures, which not only maintain efficient computation but also enhance feature representation, facilitating the model’s ability to capture multi-scale and multi-level target information. However, the core operations inside the C2f module remain stacks of standard convolutions. Standard convolutions have the property of spatial sharing and channel specificity, meaning that the same set of convolutional kernel weights slides and is shared across all spatial positions of the feature map [21]. This property weakens the model’s adaptive modeling capability with respect to the input content. The fixed kernels in standard convolutions make it difficult for the model to dynamically focus on the key regions of different vehicles, leading to insufficient feature discrimination for densely packed small targets and inadequate utilization of spatial contextual information.

**Figure 3:**
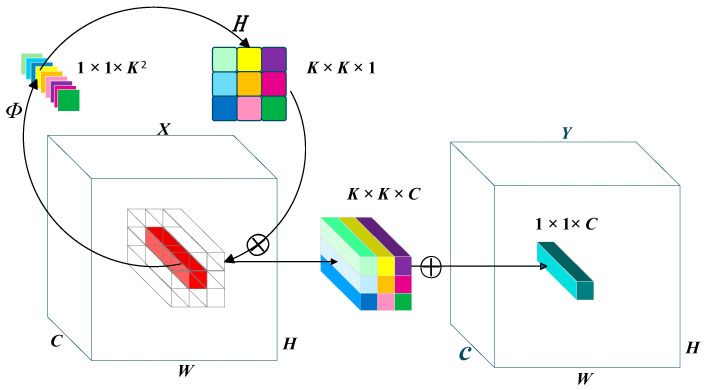
The core structure of Involution. Note: Different colored cubes indicate independent feature channels; the red block represents the sampled receptive field of involution.

Involution adopts the principle of “channel sharing and spatial specificity”, which is opposite to that of standard convolution. The core of Involution lies in the fact that the kernel weights are not fixed but are dynamically generated based on the feature content at each spatial position of the input feature map. This means that for a local region potentially containing a vehicle, Involution can generate a dedicated convolution kernel highly correlated with the content of that region, thereby achieving adaptive and highly sensitive feature extraction for vehicles of varying appearances, scales, and orientations. The core structure of Involution is shown in Figure 3.

The dynamic spatial modeling capability of Involution enables the network to adaptively focus on regions where vehicle targets may appear. This capability allows the introduction of Involution to effectively address the issues of target adhesion and missed detection. Involution inherently possesses a larger effective receptive field, enabling it to aggregate broader contextual information, which helps the model establish spatial correlations between vehicles and their surrounding environment (e.g., roads), thereby improving the robustness of detection under complex backgrounds. Moreover, because the kernels of Involution share parameters across the whole channel dimension, this operation significantly enhances spatial modeling capability while incurring extremely low parameter and computational overhead, largely satisfying the dual constraints of lightweight design and real-time performance required by UAV platforms.

The parameter count formula for a standard convolution is shown in Equation (1). Standard convolution is limited by the fact that its parameter count is proportional to the square of the number of channels, which has long restricted the kernel sizes to be 3 × 3, thereby limiting the effective receptive field of a single operation.

(1) $${Params}_{conv}=K^{2}\times C_{in}\times C_{out}$$

Assuming $C_{in}=C_{out}=C$, this simplifies the right-hand side of Equation (1) to $K^{2}\times C^{2}$. This quadratic relationship has confined the kernel size K to 3 × 3. In comparison, the parameter count of Involution comes from two 1 × 1 convolutions in the kernel generation function, as shown in Equation (2):

(2) $${Params}_{inv}=\frac{C^{2}}{r}+\frac{G\cdot K^{2}\cdot C}{r}$$

To balance enhanced feature extraction capability with model efficiency, this study introduces further improvements in the C2f module located between the two downsampling (SCDown) layers in the deep part of the YOLOv10 backbone. The core operation is to retain the overall branching structure of this C2f module while replacing the standard 3 × 3 convolutions inside some of its bottleneck structures with 7 × 7 Involution convolutions.

#### 2.2.2. Feature Fusion Network

The neck network of the YOLOv10 model performs multi-scale feature fusion through the Path Aggregation Network (PANet) structure [22]. The PANet structure transforms and integrates the concatenated multi-level features via stacked C2f modules. However, the C2f module is constrained by a fixed receptive field, making it difficult to establish long-range pixel-wise dependencies during the fusion, which in turn limits the interaction efficiency between deep semantic information and shallow details in the neck network. Moreover, the PANet structure still adopts fixed concatenation and convolution operations. This design not only weakens the dynamic discrimination capability regarding the importance of fused feature channels but also introduces background noises and information redundancy. ACmix is placed in the neck because it operates on multi-scale features (P3, P4, P5) and can simultaneously refine local textures (via its convolution path) and model long-range dependencies (via its self-attention path). This is particularly beneficial for UAV images where small vehicles require fine-grained details from shallow features, while their context (e.g., road structure) requires global semantics from deep features.

**Figure 4:**
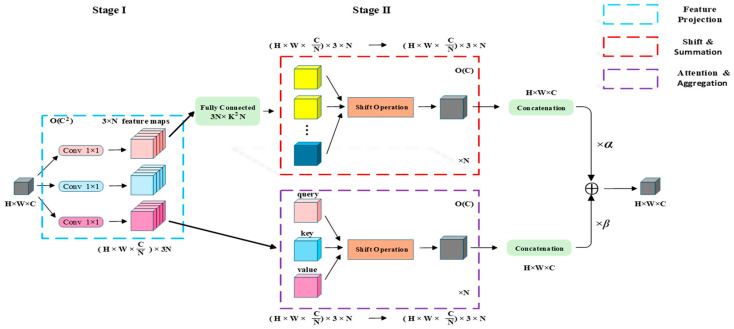
The structure of ACmix.

ACmix (Attention and Convolution Mixed) is a fusion-type feature learning operator that adopts a paradigm of “homogeneous computation and heterogeneous aggregation”, which differs from the traditional approach of simply paralleling two independent modules. The structure of ACmix is illustrated in Figure 4.

Both standard convolution and self-attention rely on the 1 × 1 convolution for feature projection, which dominates computational cost. In the second stage, convolution uses shift-and-sum for local aggregation, while self-attention uses weighted averaging for global context. As shown in Figure 4, ACmix unifies the first stage into three shared 1 × 1 convolutions, feeding intermediate features into parallel convolution and self-attention paths. Their outputs are dynamically fused by two learnable scalars. The convolution path generates kernels via a lightweight fully connected layer and employs depthwise separable convolution for efficient local reorganization. The self-attention path reshapes features into queries, keys, and values, computing pixel correlations via a similarity function. This design enables ACmix to jointly extract fine-grained local textures and global context for vehicle targets captured in UAV images with scale variation and complex backgrounds. The fused features preserve edge and contour details while suppressing background white noise, enhancing multi-scale feature fusion adaptability with negligible extra computation at cost.

#### 2.2.3. Detection Head

**Figure 5:**
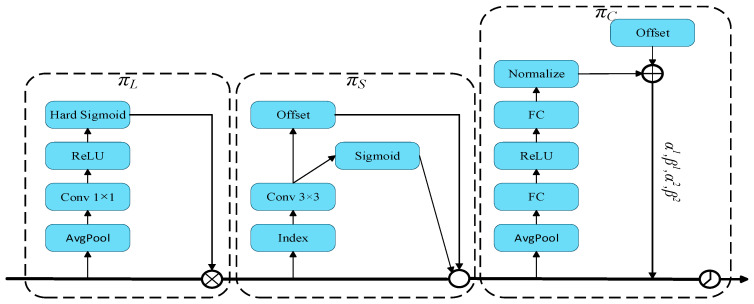
The structure of DyHead.

In the task of UAV-assisted vehicle detection, the detection head of the model serves as the terminal for feature decoding, directly determining the accuracy of object classification and bounding box localization. DyHead (Dynamic Head) is a dynamic feature-enhancement-detection head capable of adaptively adjusting feature responses at the spatial, channel, and task levels. The design of DyHead is not a simple stacking of attention mechanisms but rather a dynamic recalibration of features from three dimensions: scale, space, and task. The original YOLOv10 detection head applies static convolutions uniformly across all spatial locations and channels. In UAV images, vehicle scales vary drastically (from tens to hundreds of pixels), and occlusion patterns are irregular. DyHead is therefore used to replace the original head because its three attention mechanisms (scale, space, task) dynamically recalibrate features based on the input content. This placement is natural as the detection head is the final feature decoding stage, where adaptive weighting can most directly impact classification and regression. Replacing the original detection head of YOLOv10 with DyHead will theoretically enable the detection head to adaptively focus on key information based on the image content. The structure of DyHead is illustrated in Figure 5.

In the YOLOv10 model, the original decoupled detection head processes are featured by applying the same convolution kernels and weights across all spatial positions, channels, and tasks. Although this design improves detection efficiency by separating the classification and regression branches, it still falls into the category of static homogenization, making it difficult for the model to dynamically adjust its response according to the input content. This pattern is more prone to feature confusion and response attenuation when dealing with tiny, adherent, or partially occluded vehicles captured from high-altitude UAVs, leading to missed detections and localization errors.

DyHead employs a dynamic feature enhancement mechanism that recalibrates features through three serial dynamic enhancement units. First, to address the significant scale variation in vehicles in UAV images, scale-aware attention $\pi_{L}$ dynamically weights the feature levels, as formulated in Equation (3):

(3) $$\pi_{L}(F)\cdot F=\sigma \left(f_{conv}\left(\frac{1}{S}\sum_{S}F\right)\right)\cdot F$$

where $f_{conv}$ is 1 × 1 convolution and σ is the hard-sigmoid activation function. Scale-aware attention aggregates spatial information via global average pooling, enabling the network to adaptively enhance channels matching the target scale, thereby suppressing background noise. Second, spatial-aware attention employs deformable convolution to achieve sparse sampling and feature aggregation, as shown in Equation (4):

(4) $$\pi_{S}(F)\cdot F=\frac{1}{K}\sum_{k=1}^{K}\sum_{j=1}^{S}w_{k,j}\cdot F(j;p_{k}+\Delta p_{k})\cdot \Delta m_{k}$$

where K is the number of sampling points and $\Delta p_{k}$ and $\Delta m_{k}$ are the learned offsets and modulation scalars of the deformable convolution, respectively. Here, $w_{k,j}$ denotes the learnable weight for the k-th sampling point at the j-th spatial location, which is normalized across sampling points to sum to 1. Spatial-aware attention enables the model to focus on vehicle-dense regions, thereby improving the perception of occluded targets. Finally, task-aware attention dynamically switches channels to coordinate the two tasks, reducing classification errors and bounding box offset issues. Its formulation is given in Equation (5):

(5) $$\pi_{C}(F)\cdot F=\max\left(\alpha^{1}(F)\cdot F+\beta^{1}(F),\alpha^{2}(F)\cdot F+\beta^{2}(F)\right)$$

where α and β are learned hyper-functions that control the activation thresholds. This mechanism enables the classification path to focus on semantic discrimination while the regression path emphasizes geometric precision, reducing task conflicts.

The three attention mechanisms are combined in sequences to form the complete DyHead module, transforming the detection head from a static and uniform design into a content-aware and dynamic decoder.

In YOLOv10, the original detection head consists of two separate branches (classification and regression), each implemented as a sequence of two 3 × 3 convolutional layers followed by a 1 × 1 convolutional layer. The input feature channels to the detection head are 256 (for P3), 512 (for P4), and 1024 (for P5). In our YOLOv10-IAD, we replace the entire detection head with a single DyHead module applied to each level of the feature pyramid (P3, P4, P5). The DyHead module is configured with three sequential attention stages (scale, spatial, task), each implemented with a hidden dimension of 256. The number of deformable sampling points K in spatial-aware attention is set to 9. The total additional parameters introduced by DyHead are approximately 0.12 M, which is included in the overall parameter count (2.9 M).

## 3. Results and Discussion

### 3.1. Experimental Environments and Dataset

The experimental environment was configured as follows: the computer operating system was Ubuntu 20.04, the GPU card was an NVIDIA RTX 3090 with 24 GB memory, and the CPU card was an Intel Xeon Platinum 8362. Python version 3.8 was used, and the deep learning framework was PyTorch 2.1.0 with CUDA 12.1. All training images were uniformly resized to 640 × 640 pixels. The number of training epochs was set to 100, and the optimizer was stochastic gradient descent (SGD). The batch size was set to the maximum value allowed under the default hyperparameters. The detailed experimental environment is presented in Table 1.

To verify the generalization ability and accuracy of the model, the datasets we selected must contain dense detection targets, significant occlusion, and variable scenes. Therefore, we choose two authoritative benchmarks in this field, VisDrone2019 [27] and UAVDT [28], to effectively validate the proposed model.

VisDrone2019 contains over 10,200 static UAV images and video sequences, with more than 2.6 million annotated objects across 14 cities in China under various weather and lighting conditions. It includes over ten categories (cars, buses, trucks, etc.), and approximately 50% of low-altitude targets are smaller than 32 × 32 pixels, posing a significant challenge for small-scale detection.

UAVDT comprises about 80,000 high-resolution image frames (1080 × 540) covering diverse traffic scenes. It includes variations in weather, flight altitude, and drone motion (translation, rotation, diving), leading to severe scale changes, occlusion, and motion blur. Scenes range from highways and urban roads to intersections, with both sparse and congested traffic conditions. Table 2 provides a comparison of the different datasets seen from the public domain.

### 3.2. Evaluation Indexes

The evaluation metrics selected in this study are model parameters (Param), mean average precision (mAP), and recall (R), respectively. The model parameters (Param) serve as an indicator of whether a model is lightweight or not, and the recall (R) effectively reflects the rate of missed detections in the model. The calculation of model parameters and recall is shown in Equations (6) and (7).

(6) $$P=\frac{TP}{TP+FP}$$

(7) $$R=\frac{TP}{TP+FN}$$

In these two equations, the parameter TP (True Positives) represents the number of correct detections, FP (False Positives) represents the number of incorrect detections, and FN (False Negatives) represents the number of missed detections. Three parameters form the basis for the calculation of recall.

Mean average precision (mAP) is a metric that effectively reflects the accuracy of the model. It is composed of precision (P) and recall (R), and its calculation is shown in Equation (9). By setting different IoU thresholds (e.g., IoU at 0.5 or IoU from 0.5 to 0.95), we can obtain the values of mAP@0.5 and mAP@0.5:0.95. The specific calculation method is as follows.

(8) $$AP=\int_{0}^{1}P(R)dR$$

(9) $$mAP=\frac{1}{N}\sum_{i=1}^{N}AP_{i}$$

### 3.3. Experimental Results and Analysis

#### 3.3.1. Ablation Experiments

**Figure 6:**
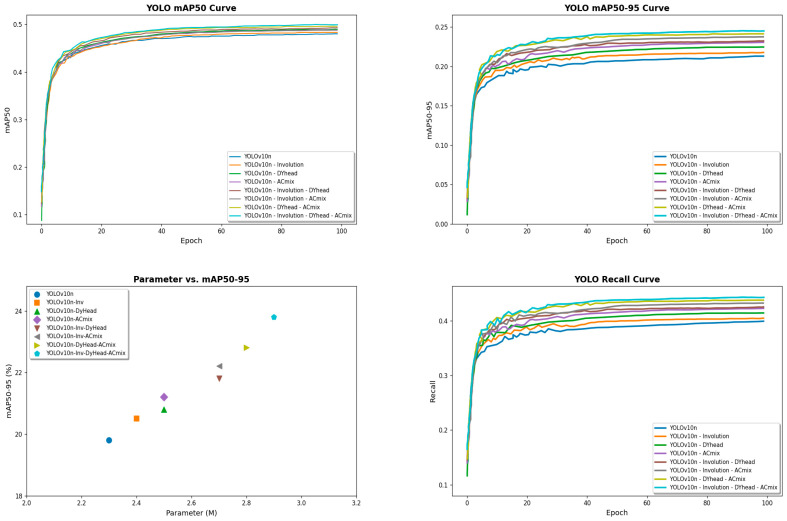
Ablation experiments with the VisDrone2019 datasets.

**Figure 7:**
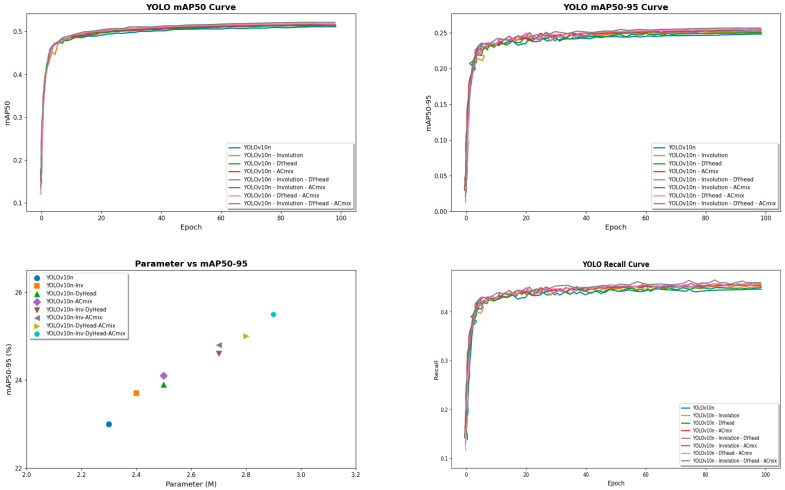
Ablation experiments with the UAVDT datasets.

Ablation experiments were conducted by progressively adding improved modules to the YOLOv10n baseline model. The experiments were performed with both the VisDrone2019 and UAVDT datasets. Results using the VisDrone2019 datasets are presented in Table 3 and Figure 6, while those using the UAVDT datasets are presented in Table 4 and Figure 7.

Ablation experiments were conducted on both the VisDrone2019 and UAVDT datasets. Using YOLOv10n as the baseline model with 2.3 million parameters and 6.7 GFLOPs, the Involution, DyHead, and ACmix modules were introduced individually first and then in combination.

On the VisDrone2019 dataset, the single-module introduction of ACmix has yielded the best performance among the three, achieving 40.5% recall and 44.9% mAP50 with 2.5 million parameters and 7.1 GFLOPs. This improvement is mainly attributed to the hybrid design of ACmix, which effectively combines convolution and self-attention to capture both local and global information. On the UAVDT dataset, ACmix alone has raised recall to 44.2% and mAP50 to 50.1% under the same parameter and computational cost.

A combination of the two has demonstrated clear synergistic effects. For example, the DyHead combined with ACmix have achieved better pairwise results on both datasets: on VisDrone2019, it reached 41.8% recall and 46.8% mAP50 with 2.8 million parameters and 7.5 GFLOPs, and on UAVDT, it attained 45.2% recall and 51.4% mAP50 under the same resource configuration. The combination of Involution and ACmix also showed complementary gains on both datasets.

While all three modules were integrated, the full YOLOv10-IAD model achieved the best overall performance. On VisDrone2019, it reached 42.5% recall, 47.2% mAP50, and 23.0% mAP50–95 with 2.9 million parameters and 7.7 GFLOPs. On UAVDT, it achieved 45.5% recall, 52.0% mAP50, and 25.3% mAP50–95 under the same resource budget. Compared to the baseline model, the improvements on VisDrone2019 were 3.1% in recall, 3.7% in mAP50, and 3.2% in mAP50–95, respectively; on UAVDT, the gains were 2.0% in recall, 3.5% in mAP50, and 2.3% in mAP50–95, respectively. The slightly improvements on UAVDT may be attributed to the more concentrated vehicle scales in that dataset, which leverage the synergy better among the three modules.

In terms of inference speed, the full YOLOv10-IAD model achieves 153 FPS on an NVIDIA RTX 3090 GPU, which is only 16% lower than the baseline YOLOv10n (182 FPS) but brings significant accuracy gains (+3.7% mAP50). This trade-off is acceptable for real-time UAV applications (30 FPS threshold). Even the slowest variant in our ablation (DyHead + ACmix) runs at 155 FPS, well above real-time requirements.

Among the three modules, ACmix alone improves mAP50 by 1.4% on VisDrone2019 (from 43.5% to 44.9%), which is higher than the gains from Involution (0.5%) and DyHead (0.8%). This is because ACmix directly addresses the fundamental bottleneck of the neck network: the inability to model long-range dependencies across scales. By fusing convolution and self-attention, ACmix enhances the feature representation at the fusion stage, which benefits all subsequent detection heads. In contrast, Involution only enhances spatial modeling in the backbone, and DyHead only adjusts the final outputs. Therefore, ACmix’s effect propagates through the entire network, yielding a larger overall impact.

In summary, each of the three modules individually improves detection performance, and their combination yields further gains while maintaining manageable computational overhead. The proposed YOLOv10-IAD model exhibits strong generalization and robustness for UAV-assisted vehicle detection on both datasets.

#### 3.3.2. Comparison and Analysis of Different Models

We compare the proposed YOLOv10-IAD with two-stage detectors (e.g., Faster R-CNN, Mask R-CNN), RT-DETR, and YOLO series on VisDrone2019 and UAVDT. All comparison models were trained for 100 epochs on the same training set under identical experimental settings (same optimizer, learning rate, batch size, and input resolution of 640 × 640). The results are shown in Table 5 and Table 6.

YOLOv10-IAD strikes an excellent accuracy–efficiency trade-off for UAV-assisted vehicle detection. It outperforms two-stage detectors with <1/14 the parameters and achieves higher mAP50 than the DETR series while using only 1/6 the parameters and fewer FLOPs, and it surpasses lightweight YOLO models by 3.5–15 pp (mAP50), 2–5 pp (mAP50–95), and 2–6 pp (recall) while adding only 0.6 M parameters. These test results have confirmed the efficacy of Involution, ACmix, and DyHead, offering a lightweight high-performance solution for onboard real-time detection.

#### 3.3.3. Performance Analysis on Samples of Different Difficulty

**Figure 8:**
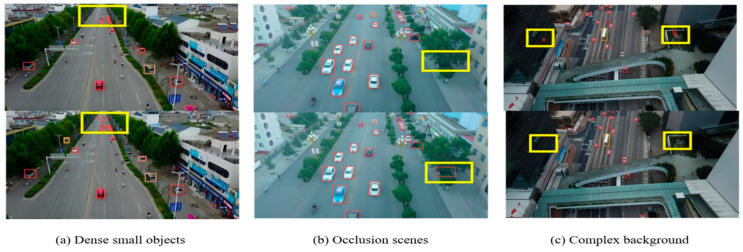
Detection comparison in different scenes.

To evaluate the robustness of YOLOv10-IAD in complex scenes, three representative sample types were selected from the VisDrone2019 validation dataset: dense small-target, partially occluded, and complex-background scenes. Figure 8 compares detection results of the baseline YOLOv10n and the proposed YOLOv10-IAD model in these scenarios.

From Figure 8a, an intersection scene with many small vehicles, the baseline YOLOv10n missed multiple targets, especially distant small ones, while YOLOv10-IAD successfully detected them. This improvement is attributed to the 7 × 7 kernel of the Involution module, which aggregates contextual information via channel sharing to compensate for the lack of small-target pixel details, and the ACmix module, which uses global attention to distinguish adjacent vehicle boundaries. In Figure 8b, when vehicles were partially occluded by trees or traffic signs, the baseline model lost the targets entirely, whereas YOLOv10-IAD correctly detected them. This improvement comes from the task-aware decoupling mechanism of DyHead, which enables the regression branch to infer complete contours from locally visible regions while maintaining high semantic confidence in the classification branch, thereby improving detection accuracy for occluded targets. In Figure 8c, under complex backgrounds with building shadows, road markings, and vegetation, the baseline model misclassified shadows and signs as vehicles, but YOLOv10-IAD produced no false positives. This is mainly due to the ACmix module, which fuses convolution and self-attention to suppress background noise and focuses on the model’s attention to vehicle bodies. The qualitative analysis above demonstrates that the three improved modules implemented in YOLOv10-IAD play essential roles in addressing typical challenges in UAV-assisted vehicle detection.

In addition to the representative examples in Figure 8, we further analyze failure cases under challenging conditions.

**Figure 9:**
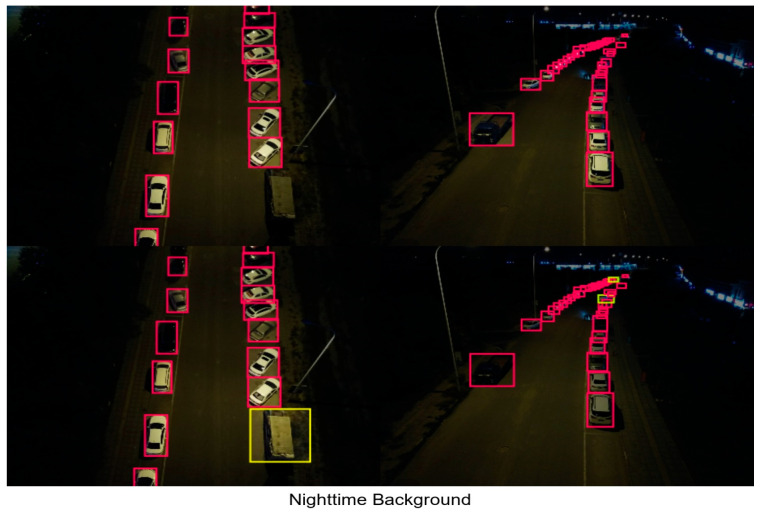
Failure case in low-light (nighttime) scenario.

**Figure 10:**
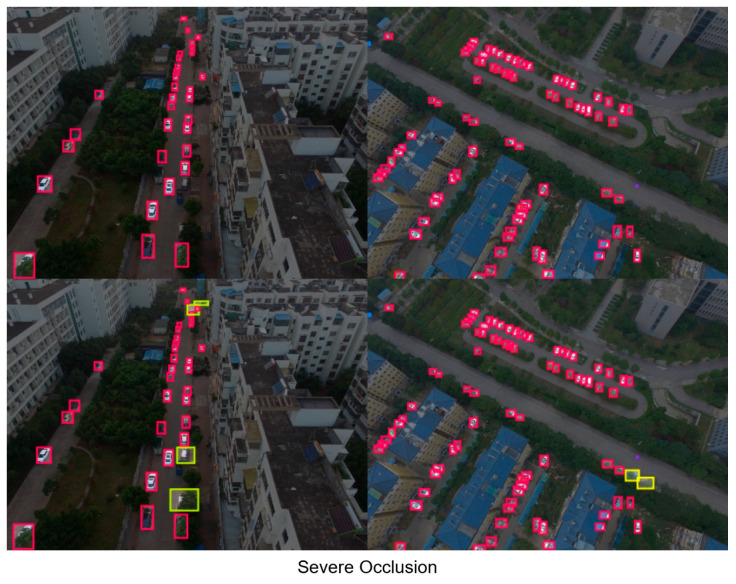
Failure case under severe occlusion.

Figure 9 demonstrates a typical failure scenario under extremely low-light conditions (night). YOLOv10-IAD fails to detect several vehicles that are barely visible to the human eye, resulting in missed detection (false negatives) of approximately 5% for small targets. Figure 10 presents a severe occlusion background, where vehicles are almost entirely covered by a tree canopy. In this case, YOLOv10-IAD model completely misses the occluded vehicle, indicating that even with the proposed DyHead module, extreme occlusion remains a challenging problem.

## 4. Conclusions

This paper focuses on vehicle detection using UAV aerial imagery, which suffers from small target sizes, dense distribution, frequent occlusions, and complex backgrounds. To address the limitations of YOLOv10 in feature extraction, multi-scale fusion, and detection head adaptability, an improved model named YOLOv10-IAD is proposed. Three new modules are proposed and integrated. Involution replaces partial standard convolutions with dynamic space-specific kernels to enlarge the receptive field and enhance spatial perception. ACmix fuses convolution and self-attention to combine local details with global context for better multi-scale feature interaction. DyHead dynamically recalibrates features through scale-, spatial-, and task-aware attention, improving localization accuracy for occluded and scale-variant targets. Ablation and comparative experiments on VisDrone2019 and UAVDT datasets have shown that each module contributes to performance gains, and their combination yields a strong synergistic effect. The full YOLOv10-IAD model significantly outperforms two-stage detectors (Faster R-CNN, Mask R-CNN), RT-DETR, and lightweight YOLO series in both accuracy and efficiency. In summary, YOLOv10-IAD achieves an excellent balance between detection accuracy and computational efficiency, providing a high-performance lightweight solution for onboard UAV vehicle detection. Future work will focus on improving dataset annotation quality and exploring more efficient network architectures.

Although the proposed YOLOv10-IAD model achieves competitive performance in UAV-assisted vehicle detection, some limitations remain. For example, due to incomplete annotations in the VisDrone2019 and UAVDT datasets, the model may still suffer from false positives or missed detections in certain scenarios. In future work, we plan to improve annotation quality through semi-supervised learning or active learning strategies. Additionally, we intend to explore more efficient network architectures, such as integrating lightweight attention mechanisms or designing adaptive multi-scale feature fusion modules specifically for tiny and occluded vehicles, to further enhance detection accuracy and robustness.

## Figures and Tables

**Table 1:** Experimental platform.

Environment	Configuration
Operating System	Ubuntu 20.04
GPU	NVIDIA RTX 3090 (24 GB)
CPU	Intel Xeon Platinum 8362
Python	3.8.19
Torch Deep Learning Framework	Torch 2.1.0 + CUDA 12.1

**Table 2:** UAV Vehicle Detection Datasets.

Datasets	Number of Images	Target Instances	Target Instance
CARPK	1448	89,777	62
AU-AIR	32,823	132,000+	4
DroneVehicle	56,878	260,000+	4.6
VEDAI	1272	3500	2.8
VisDrone2019	10,209	540,000+	50
UAVDT	80,000	840,000+	10.5

**Table 3:** Ablation experiments with the VisDrone2019 datasets.

Baseline	Involution	DyHead	ACmix	Params (M)	Flops (G)	R (%)	mAP50 (%)	mAP50–95 (%)	FPS
YOLOv10n				2.3	6.7	39.4	43.5	19.8	182
	√			2.4	6.9	40.1	44.0	20.5	178
		√		2.5	7.0	40.2	44.3	20.8	169
			√	2.5	7.1	40.5	44.9	21.2	166
	√	√		2.7	7.3	41.2	45.6	21.8	162
	√		√	2.7	7.4	41.5	46.4	22.2	158
		√	√	2.8	7.5	41.8	46.8	22.8	155
	√	√	√	2.9	7.7	42.5	47.2	23.0	153

**Table 4:** Ablation experiments with the UAVDT datasets.

Baseline	Involution	DyHead	ACmix	Params (M)	Flops (G)	R (%)	mAP50 (%)	mAP50–95 (%)	FPS
YOLOv10n				2.3	6.7	43.5	48.5	23.0	182
	√			2.4	6.9	43.8	49.5	23.7	178
		√		2.5	7.0	44.0	49.8	23.9	169
			√	2.5	7.1	44.2	50.1	24.1	166
	√	√		2.7	7.3	44.8	50.8	24.6	162
	√		√	2.7	7.4	45.0	51.1	24.8	158
		√	√	2.8	7.5	45.2	51.4	25.0	155
	√	√	√	2.9	7.7	45.5	52.0	25.3	153

**Table 5:** Comparative experiments on the VisDrone2019 datasets.

Method	Params (M)	Flops (G)	R (%)	mAP50 (%)	mAP50–95 (%)
Faster-R-CNN	41.34	190	36.5	38.2	16.8
Mask-R-CNN	43.99	243	37.2	39.0	17.2
RT-DETR	19.88	35.2	38.0	40.5	18.1
YOLOv5n	1.76	4.1	29.5	32.8	18.3
YOLOv5s	7.05	7.96	30.0	30.4	17.5
YOLOv8n	3.2	8.1	30.2	32.4	18.9
YOLOv8s	11.1	28.4	35.0	39.2	21.0
YOLOv10n (Baseline)	2.3	6.7	39.4	43.5	19.8
YOLOv11n	2.58	6.3	33.5	34.5	20.0
YOLOv11s	9.41	21.5	36.5	39.1	23.4
YOLOv10-IAD (Present)	2.9	7.7	42.5	47.2	23.0

**Table 6:** Comparative experiments on the UAVDT datasets.

Method	Params (M)	Flops (G)	R (%)	mAP50 (%)	mAP50–95 (%)
Faster-R-CNN	41.34	190	37.2	38.5	20.2
Mask-R-CNN	43.99	243	38.1	39.2	20.8
RTDETR	19.88	59.85	39.0	41.0	21.5
YOLOv5n	1.76	4.1	38.0	42.5	22.0
YOLOv5s	7.05	7.96	39.5	44.0	22.8
YOLOv8n	3.2	8.1	38.5	43.2	22.5
YOLOv8s	11.1	28.4	41.0	45.5	23.8
YOLOv10n (Baseline)	2.3	6.7	43.5	48.5	23.0
YOLOv11n	2.58	6.3	42.0	47.0	24.0
YOLOv11s	9.41	21.5	43.2	48.2	24.5
YOLOv10-IAD (Present)	2.9	7.7	45.5	52.0	25.3

## Data Availability

You can access the VisDrone2019 and UAVDT datasets through the following links. VisDrone2019 dataset: https://github.com/VisDrone/VisDrone-Dataset (accessed on 15 October 2025) UAVDT dataset: https://sites.google.com/view/grli-uavdt/ (accessed on 26 October 2025).

## References

[1] Yang J. (2021). Research on Vehicle Target Detection Algorithm from UAV Perspective. Ph.D. Thesis.

[2] Telikani A., Sarkar A., Du B., Shen J. (2024). Machine Learning for UAV-Aided ITS: A Review with Comparative Study. IEEE Trans. Intell. Transp. Syst..

[3] Rahman M.H., Sejan M.A.S., Aziz M.A., Tabassum R., Baik J.I., Song H.K. (2024). A Comprehensive Survey of Unmanned Aerial Vehicles Detection and Classification Using Machine Learning Approach: Challenges, Solutions, and Future Directions. Remote Sens..

[4] Nikouei M., Baroutian B., Nabavi S., Taraghi F., Aghaei A., Sajedi A., Moghaddam M.E. (2025). Small Object Detection: A Comprehensive Survey on Challenges, Techniques and Real-World Applications. Intell. Syst. Appl..

[5] Hua W., Chen Q. (2025). A Survey of Small Object Detection Based on Deep Learning in Aerial Images. Artif. Intell. Rev..

[6] Girshick R., Donahue J., Darrell T., Malik J. (2014). Rich Feature Hierarchies for Accurate Object Detection and Semantic Segmentation. Proceedings of the IEEE Conference on Computer Vision and Pattern Recognition (CVPR), Columbus, OH, USA, 23–28 June 2014.

[7] Redmon J., Divvala S., Girshick R., Farhadi A. (2016). You Only Look Once: Unified, Real-Time Object Detection. Proceedings of the IEEE Conference on Computer Vision and Pattern Recognition (CVPR), Las Vegas, NV, USA, 27–30 June 2016.

[8] Ren S., He K., Girshick R., Sun J. (2017). Faster R-CNN: Towards Real-Time Object Detection with Region Proposal Networks. IEEE Trans. Pattern Anal. Mach. Intell..

[9] Lin T.Y., Dollár P., Girshick R., He K., Hariharan B., Belongie S. (2017). Feature Pyramid Networks for Object Detection. Proceedings of the IEEE Conference on Computer Vision and Pattern Recognition (CVPR), Honolulu, HI, USA, 21–26 July 2017.

[10] Hansen K.S., Bruun F.M., Sermsar F., Nygaard M., Koca M. (2024). Comparative Analysis of SSD and Faster R-CNN in UAV-Based Vehicle Detection. Proceedings of the 2024 8th International Artificial Intelligence and Data Processing Symposium (IDAP), Malatya, Turkey, 21–22 September 2024.

[11] Ghasemi Darehnaei Z., Shokouhifar M., Mirhosseini S.M., Yazdanjouei H. (2022). Two-Stage Swarm Intelligence Ensemble Deep Transfer Learning (SI-EDTL) for Vehicle Detection Using Unmanned Aerial Vehicles. Concurr. Comput. Pract. Exp..

[12] Liu W., Anguelov D., Erhan D., Szegedy C., Reed S., Fu C.Y., Berg A.C. (2016). SSD: Single Shot MultiBox Detector. Proceedings of the European Conference on Computer Vision (ECCV), Amsterdam, The Netherlands, 11–14 October 2016.

[13] Kang J., Yang H., Kim H. (2025). Simplifying Two-Stage Object Detectors for On-Board Remote Sensing. IEEE Access.

[14] Bochkovskiy A., Wang C.Y., Liao H.Y.M. (2020). YOLOv4: Optimal Speed and Accuracy of Object Detection. arXiv.

[15] Ge Z., Liu S., Wang F., Li Z., Sun J. (2021). YOLOX: Exceeding YOLO Series in 2021. arXiv.

[16] Xiang Y., Li B., Wan T. (2025). Vehicle Detection Algorithm for UAV Aerial Photography Based on Improved YOLOv5. Comput. Meas. Control.

[17] Zhu L., Xiong J., Xiong F., Hu H., Jiang Z. (2023). YOLO-Drone: Airborne Real-Time Detection of Dense Small Objects from High-Altitude Perspective. arXiv.

[18] Cao J., Qiao G., Chen M., Zou X., Liu D. (2024). Improvement Strategy of YOLO Algorithm for Small Target Detection from High-Altitude View. J. Comput. Appl..

[19] Zhou Y., Wang L., Zhang H., Huo J. (2025). Vehicle Detection Method Based on Multi-Layer Selective Feature for UAV Aerial Images. J. King Saud. Univ.—Comput. Inf. Sci..

[20] Wang A., Chen H., Liu L., Chen K., Lin Z., Han J., Ding G. (2024). YOLOv10: Real-Time End-to-End Object Detection. arXiv.

[21] Krizhevsky A., Sutskever I., Hinton G.E. (2012). ImageNet Classification with Deep Convolutional Neural Networks. Proceedings of the Advances in Neural Information Processing Systems (NeurIPS), Lake Tahoe, NV, USA, 3–6 December 2012.

[22] Liu S., Qi L., Qin H., Shi J., Jia J. (2018). Path Aggregation Network for Instance Segmentation. Proceedings of the IEEE Conference on Computer Vision and Pattern Recognition (CVPR), Salt Lake City, UT, USA, 18–23 June 2018.

[23] Wang C.Y., Bochkovskiy A., Liao H.Y.M. (2023). YOLOv7: Trainable Bag-of-Freebies Sets New State-of-the-Art for Real-Time Object Detectors. Proceedings of the IEEE/CVF Conference on Computer Vision and Pattern Recognition (CVPR), Vancouver, BC, Canada, 18–22 June 2023.

[24] Li D., Hu J., Wang C., Li X., She Q., Zhu L., Zhang T., Chen Q. (2021). Involution: Inverting the Inherence of Convolution for Visual Recognition. Proceedings of the IEEE/CVF Conference on Computer Vision and Pattern Recognition (CVPR), Nashville, TN, USA, 20–25 June 2021.

[25] Pan X., Ge C., Lu R., Song S., Chen G., Huang Z., Huang G. (2022). On the Integration of Self-Attention and Convolution. Proceedings of the IEEE/CVF Conference on Computer Vision and Pattern Recognition (CVPR), New Orleans, LA, USA, 19–24 June 2022.

[26] Dai X., Chen Y., Xiao B., Chen D., Liu M., Yuan L., Zhang L. (2021). Dynamic Head: Unifying Object Detection Heads with Attentions. Proceedings of the IEEE/CVF Conference on Computer Vision and Pattern Recognition (CVPR), Nashville, TN, USA, 20–25 June 2021.

[27] Zhu P., Wen L., Du D., Bian X., Ling H., Hu Q., Nie Q., Cheng H., Liu C., Liu X. (2018). VisDrone: The Vision Meets Drone Object Detection in Image/Video Challenge Results. Proceedings of the European Conference on Computer Vision (ECCV) Workshops, Munich, Germany, 8–14 September 2018.

[28] Du D., Qi Y., Yu H., Yang Y., Duan K., Li G., Zhang W., Huang Q., Tian Q. (2018). The Unmanned Aerial Vehicle Benchmark: Object Detection and Tracking. Proceedings of the European Conference on Computer Vision (ECCV), Munich, Germany, 8–14 September 2018.

